# Cystic degeneration of hepatic adenoma: a rare complication of hepatic adenoma

**DOI:** 10.1259/bjrcr.20170056

**Published:** 2017-11-17

**Authors:** Sin Yee Foo, Laura Paul, Subra Viswanathan

**Affiliations:** ^1^Radiology, ST4 Radiology, West of Scotland Deanery, Glasgow, Scotland; ^2^Radiology, ST2 Radiology, West of Scotland Deanery, Glasgow, Scotland; ^3^Glasgow Royal Infirmary, Glasgow, Scotland

## Abstract

This case report describes a rare complication of hepatic adenomata in a 33-year-old female. The patient initially presented with abdominal pain, and baseline imaging demonstrated several hepatic adenomas, the largest of which (approximately 8 cm) was adjacent to the inferior vena cava. Owing to the location of this adenoma, surgical/vascular intervention was deemed inappropriate. The patient was actively observed for approximately 4 years, and managed supportively during any recurrent episodes. With follow-up CT/MRI scans, the “natural history” of this particular lesion, including haemorrhage, thrombosis and infarction, was observed. However, as intervention was unsuitable, further MRI was performed in view of these complications, allowing observation of the end-stage features of the adenoma. Appearances were consistent with a rare complication of hepatic adenoma, *i.e*. cystic degeneration, a process well documented in uterine leiomyoma.

## Clinical presentation

HB, a 33-year-old female, first presented in November 2012 with severe right upper quadrant pain. She was on the oral contraceptive pill at the time. Baseline ultrasound, CT and MRI scans demonstrated multiple liver lesions with features in keeping with hepatic adenomata. The patient re-presented 4 weeks later, and repeat imaging revealed likely infarction of the largest lesion.

Owing to the location of the dominant adenoma, resection/intervention was not advisable and HB was closely monitored by the regional liver unit and local hepatology team.

Over the next few months, the patient generally improved and follow-up MRI was performed at 10 months from initial presentation, which demonstrated slight involution of the infarcted adenoma.

For the next 3 years, she was followed up annually by the local hepatology team.

In August 2016, an episode of acute epigastric pain with newly deranged liver biochemistry prompted a review by the local hepatologist and an updated MRI.

## Differential diagnosis

Fibrolamellar carcinoma

Hepatocellular carcinoma

Metastases

## Investigations/Imaging findings

**Figure 1.**
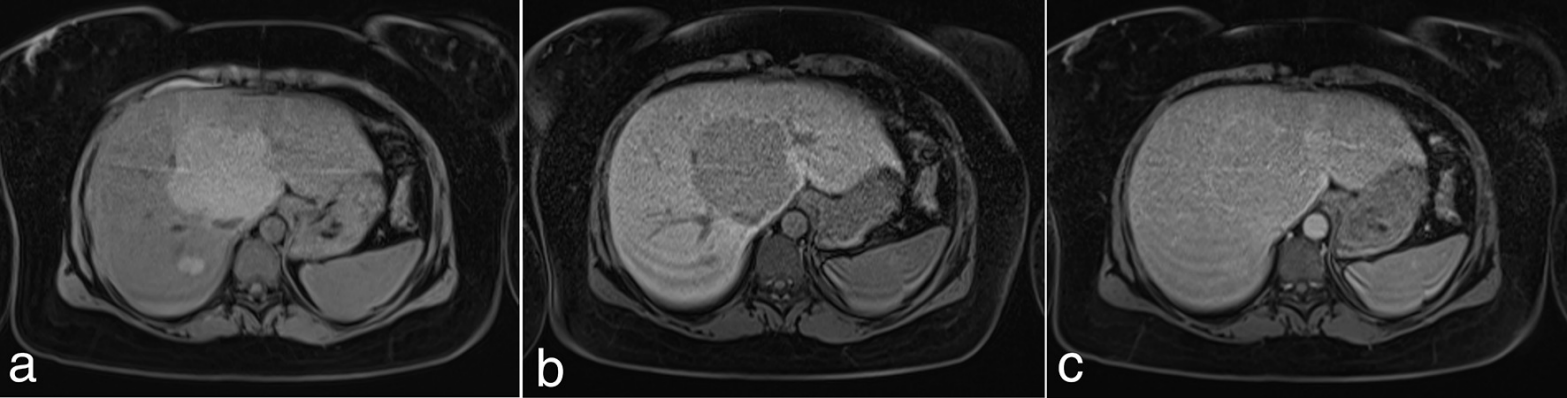
Dynamic MRI August 2012. (a) Pre-Primovist (b) Hepatobiliary phase (c) Equilibrium phase displaying pseudocapsule appearance.

Presentation ultrasound, CT and MRI in August 2012 showed several hepatic adenomata. The largest adenoma measured approximately 8 cm and arose from the caudate lobe abutting the inferior vena cava and hepatic veins. On *T*_2_ fat-saturated MRI the lesions were isointense and diffusion-weighted imaging was suggestive of benign aetiology; dynamic sequences showed diffuse arterial enhancement and prompt washout with pseudocapsule appearance (Figure 1).

On portal venous phase CT 4 weeks later, the dominant adenoma showed no enhancement (previously enhancing) and features in keeping with infarction.

**Figure 2.**
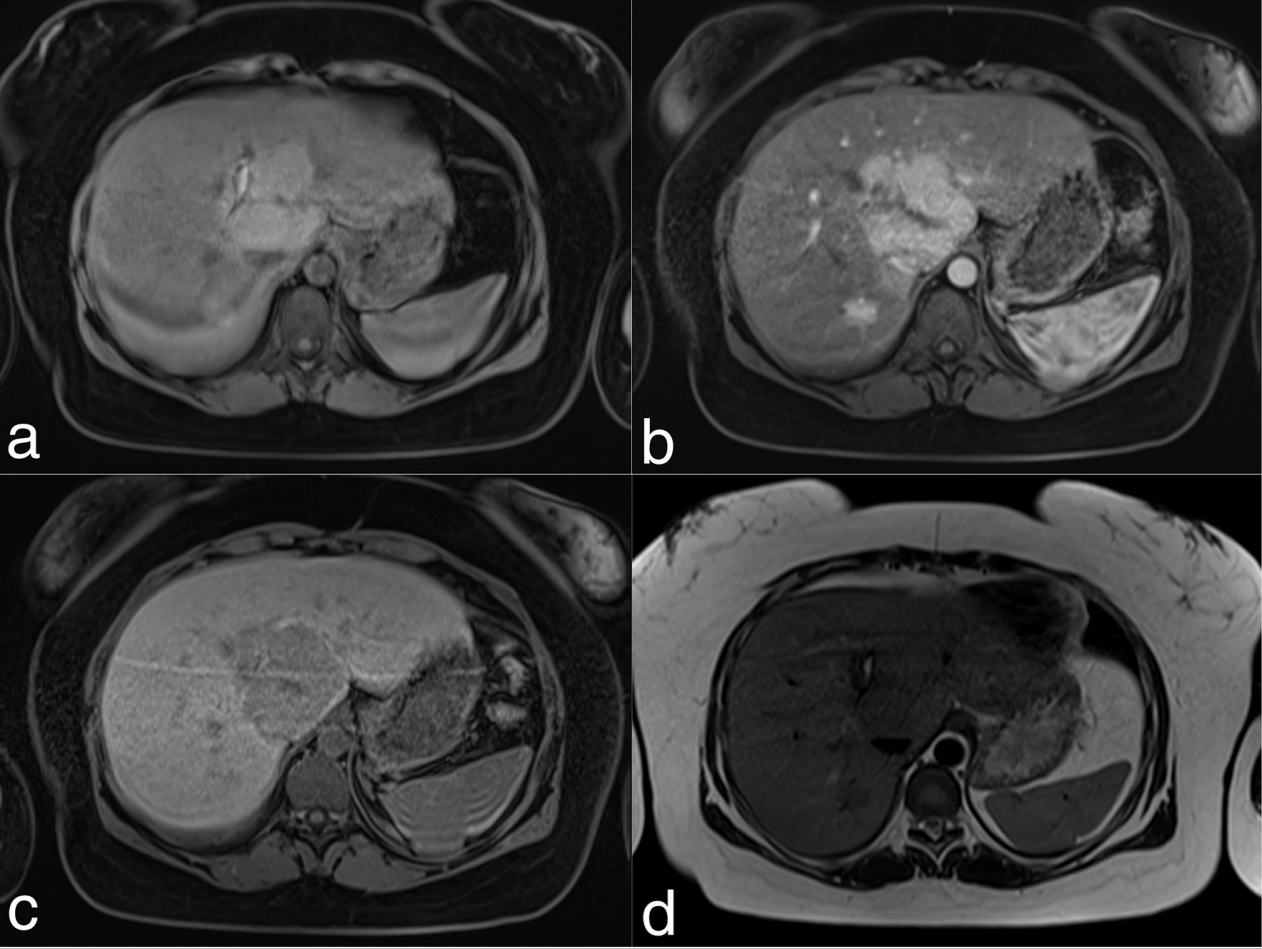
Follow-up imaging in April 2013. (a) Pre-contrast, (b) arterial and (c) hepatobiliary phase *T*_1_ weighted sequences (d) *T*_2_ SPAIR sequence. These sequences demonstrate involution of the dominant lesions with altered haemoglobin signal and reduction in size.

Interval follow-up MRI during the periods of April 2013 to September 2015 showed essentially stable disease, with slight involution of the infarcted adenoma (Figure 2).

**Figure 3.**
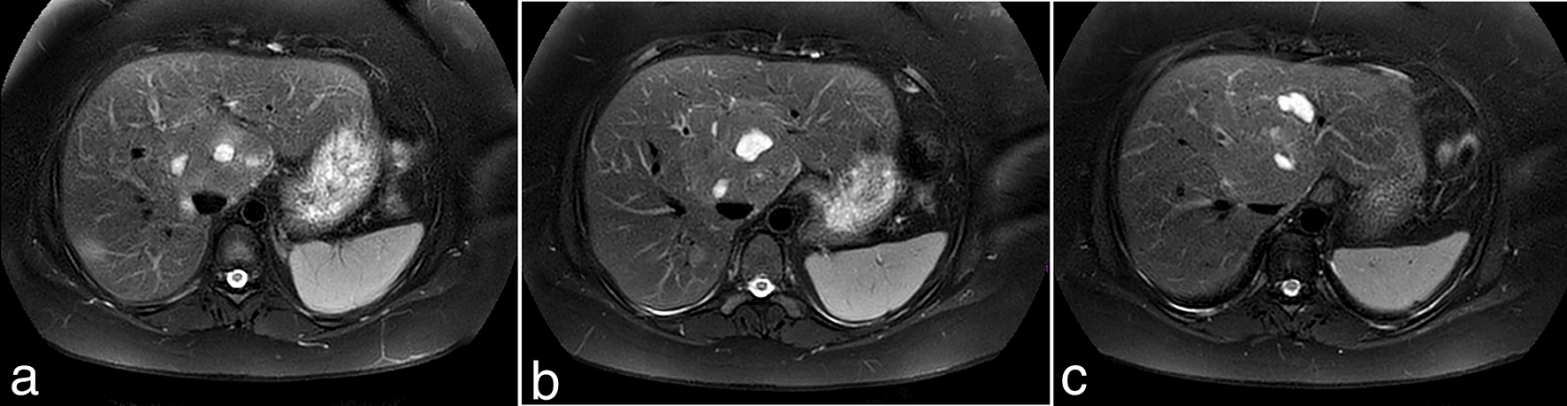
Consecutive slice through the liver on follow-up MRI (*T*_2_ SPAIR) in September 2016 demonstrates multifocal high signal within the dominant adenoma.

**Figure 4.**
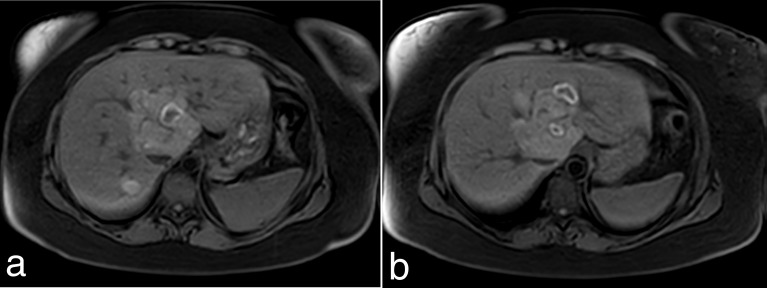
MRI September 2016. Consecutive slice pre-gadolinium *T*_1_ FATSAT through the liver demonstrates high signal rim around the high *T*_2_ SPAIR signal lesions.

A single phase (portal venous) CT was performed when the patient presented with an acute episode of pain in August 2016 while on holiday, which demonstrated an increase in size and heterogeneous enhancement of the previously infarcted adenoma suggestive of recent haemorrhage or further infarction. Follow-up MRI (Figures 3, 4) performed on the patient’s return 4 weeks later revealed multiple non-enhancing cystic lesions with a peripheral high signal rim within this adenoma.

## Treatment

Supportive management including antibiotics was given (there were concerns about superadded infection following haemorrhage).

## Outcome, follow-up and discussion

Following discussion with the regional liver unit and the local team, the largest adenoma was still not felt amenable to intervention and the patient is to be managed with active observation.

## Discussion

Hepatic adenomas are benign tumours of the liver, derived from the monoclonal proliferation of hepatocytes. There are four molecular pathological subtypes of hepatic adenoma (HA). These are (1) HAs with inactivating mutations of hepatocyte nuclear factor 1α (HNF1A; HA-H), (2) HAs with activating mutations of β-catenin gene (HA-B), (3) HAs without mutations of the HNF1A or β-catenin genes and with inflammatory features (HA-I) and (4) unclassified HAs that have no specific gene mutations or unique morphologic features (HA-U).^1^ Unfortunately, in the case of HB tissue samples were not available to facilitate classification, owing to the location of the lesion.

MR imaging appearances of HAs are variable and non- specific.^2,3^ Typically, in an uncomplicated hepatic adenoma, MR imaging demonstrates a well-defined isointense or slightly hyperintense lesion on *T*_2_ weighted images and heterogeneous signal on *T*_1_ weighted images.^4^ With gadolinium, the lesion enhances markedly and homogeneously during arterial phase with complete washout during the portal phase.^5^ Approximately 4–7% of adenomata contain fat, which is echogenic on ultrasound (mimicking haemangioma), low attenuation on CT and high signal on *T*_1 _weighted images.

Complications arise when the adenomata outgrow their blood supply and can include rupture, haemorrhage, thrombosis, infarction and malignant transformation.^5^ These are well documented and described in textbooks and literature. A very rarely described phenomenon is that of cystic degeneration.^6^

Conventionally, adenomatous disease is surgically resected owing to the rare potential for malignant transformation and risk of the above complications. Recent literature suggests the possibility of treatment with radiofrequency ablation (RFA),^7^ but this requires strict patient selection criteria. Based on these criteria, HB would have been an unsuitable candidate because of the size of the largest adenoma and its location adjacent to the inferior vena cava.^8^ Resection of adenomatous disease is further contraindicated by the presence of concurrent background fatty liver and multifocality.

Transarterial embolization (TAE) has not been prospectively investigated as a means of treatment for cases where surgical resection and RFA are contraindicated. Research published in 2009 indicated that watchful waiting with TAE during episodes of haemorrhagic complication may be the best treatment option for this group of patients at present.^9^

The initial MR imaging of HB revealed liver lesions consistent with hepatic adenomas, and later demonstrated some of the more conventional sequelae including haemorrhage and infarction. However, the most recent MR imaging had features consistent with a rarely described consequence of haemorrhagic/infarcted hepatic adenoma—cystic degeneration.

The MRI features of cystic degeneration in hepatic adenomas are not well documented and scarcely found on searching the literature. A search for cystic degeneration of tumours in other anatomical locations, *e.g*. uterus, was therefore performed. Cystic degeneration is a form of degeneration that occurs following oedema, *e.g*. following haemorrhage and necrosis. The regions of oedema result in the formation of cysts with walls.^10^ Key features of uterine cystic degeneration include high signal intensity on *T*_2_ weighted images (with corresponding low T1w signal) that do not enhance with gadolinium.^10,11^ These appearances were demonstrated on HB’s most recent MRI, and although rare, would be explained by a process of cystic degeneration following the haemorrhage/infarction of a large hepatic adenoma.

## Learning points

1. Imaging characteristics are different for different subtypes of hepatic adenoma.
2. Haemorrhage or infarction of hepatic adenomas can result in the rare complication of cystic degeneration. MRI features appear similar to cystic degeneration elsewhere.
3. Surgical resection and radiofrequency ablation remain the mainstay of treatment for hepatic adenoma provided the patient meets the selection criteria.

## Consent

Written informed consent for the case to be published (including images, case history and data) was obtained from the patient for publication of this case report, including accompanying images.
